# An umbrella review of systematic reviews and meta-analyses evaluating the efficacy and safety of mechanical thrombectomy for acute ischemic stroke

**DOI:** 10.1097/MS9.0000000000005331

**Published:** 2026-07-24

**Authors:** Nicholas Aderinto, Thomas Oluwafiponmile Oyediran, Aderinola Halimat Abioye, Happiness Chisom Ezeakunne, Temitomi Jane Oyedele

**Affiliations:** a College of Health Sciences, LAUTECH Teaching Hospital, Ogbomoso, Nigeria; b College of Medicine, University of Ibadan, Ibadan, Nigeria; c College of Health Sciences, Bowen University, Iwo, Nigeria

**Keywords:** acute ischemic stroke, functional outcome, large vessel occlusion, mechanical thrombectomy, umbrella review

## Abstract

Mechanical thrombectomy (MT) is an established treatment for acute ischemic stroke (AIS) due to large vessel occlusion. However, evidence from multiple systematic reviews and meta-analyses varies across patient populations and clinical subgroups. This umbrella review aimed to synthesize and critically appraise the existing evidence on the efficacy and safety of MT in AIS. An umbrella review was conducted in accordance with PRISMA 2020 guidelines. PubMed/MEDLINE, Embase, Web of Science, and the Cochrane Database of Systematic Reviews were searched from inception to December 2025. Eligible reviews evaluated MT, with or without intravenous thrombolysis (IVT), in adults with AIS and reported outcomes including functional independence, mortality, recanalization, or hemorrhagic complications. Methodological quality was assessed using the AMSTAR-2 tool. Ten systematic reviews and meta-analyses published between 2015 and 2025 were included and were rated as high-to-moderate methodological quality. MT was consistently associated with improved functional independence and higher recanalization rates compared with standard medical therapy or IVT alone. Mortality outcomes were heterogeneous, with some reviews demonstrating reduced mortality and others showing no significant difference. Safety analyses generally showed no significant increase in symptomatic intracranial hemorrhage, although increased hemorrhagic risk was reported in certain populations, including anticoagulated and thrombocytopenic patients. Overall, current evidence from high- to moderate-quality systematic reviews supports the efficacy of MT in improving functional outcomes and recanalization in AIS. However, this umbrella review identifies persistent uncertainty across key clinical subgroups and highlights inconsistencies in mortality and safety outcomes.

## Introduction

Stroke remains a leading cause of mortality worldwide and a major contributor to disability-adjusted life years^[^1^]^. Approximately 87% of all strokes are ischemic, and in 2020 ischemic stroke accounted for nearly 49% of stroke-related deaths globally^[^2,3^]^. Major risk factors include hypertension, hypercholesterolemia, atrial fibrillation, diabetes mellitus, smoking, and alcohol use, with effective control of modifiable risk factors shown to significantly reduce stroke incidence^[^3^]^.

HIGHLIGHTS

- **Comprehensive synthesis**: First umbrella review summarizing evidence from multiple systematic reviews and meta-analyses on mechanical thrombectomy for acute ischemic stroke.
- **Efficacy and safety outcomes**: Evaluates functional independence, mortality, symptomatic intracranial hemorrhage, and procedural complications across studies.
- **Clinical guidance**: Provides high-level evidence to support informed decision-making and identifies gaps for future stroke research.

Large vessel occlusion (LVO) is a severe subtype of ischemic stroke characterized by the obstruction of major proximal cerebral arteries and accounts for approximately 10–46% of ischemic stroke cases, depending on the definition applied and the arterial segments included^[^2,4^]^. Compared with non-LVO ischemic strokes, LVO is associated with substantially higher rates of mortality and long-term disability^[^2^]^.

Rapid restoration of cerebral blood flow remains the primary goal of acute ischemic stroke (AIS) management. Intravenous recombinant tissue plasminogen activator (IV rtPA) is recommended within 4.5 hours of symptom onset^[^4^]^. However, its effectiveness is highly time-dependent and limited by several contraindications^[^4^]^. In addition, IV rtPA demonstrates reduced efficacy in proximal LVO, and delayed or incomplete reperfusion may lead to poor clinical outcomes^[^5^]^.

Mechanical thrombectomy (MT) has emerged as the standard of care for eligible patients with AIS due to LVO. By mechanically removing the occluding thrombus using endovascular devices, MT achieves higher recanalization rates and improved functional outcomes compared with medical therapy alone^[^4^]^. Despite its proven efficacy, MT remains associated with potential complications, including hemorrhagic transformation, which may contribute to increased morbidity and mortality^[^5^]^.

Numerous systematic reviews and meta-analyses have evaluated the efficacy and safety of MT across different patient populations and clinical settings. However, the findings of these reviews have not been comprehensively synthesized, making it difficult to identify areas of consensus, uncertainty, and evidence gaps. Therefore, an umbrella review is warranted to provide a higher-level overview of the available evidence.

This umbrella review aims to systematically evaluate and summarize evidence from existing systematic reviews and meta-analyses regarding the efficacy and safety of MT in AIS. This review addresses the following research questions: (1) What is the overall evidence supporting the efficacy and safety of MT in AIS? (2) How do outcomes vary across clinically relevant patient subgroups? and (3) What evidence gaps and future research priorities can be identified from the current literature? This review adheres to the TITAN guidelines^[^6^]^.

## Methods

### Study design

This umbrella review synthesized evidence from published systematic reviews and meta-analyses evaluating the efficacy and safety of MT in AIS. The review was conducted according to established methodological guidance for umbrella reviews and reported in accordance with the Preferred Reporting Items for Systematic Reviews and Meta-Analyses (PRISMA) 2020 statement. The review sought to address three questions: (1) What is the overall evidence supporting MT in AIS? (2) How do outcomes vary across clinically relevant subgroups? and (3) What evidence gaps remain in the current literature?

### Eligibility criteria

#### Inclusion criteria

Systematic reviews, with or without meta-analysis.

Systematic reviews evaluating MT in AIS.

Systematic reviews, including randomized controlled trials (RCTs), observational studies, or both.

Systematic reviews reporting at least one outcome of interest, including functional independence, mortality, recanalization, or hemorrhagic complications.

Peer-reviewed publications are published in English.

#### Exclusion criteria

Narrative reviews, editorials, commentaries, conference abstracts, and protocols.

Reviews focusing exclusively on pediatric stroke or hemorrhagic stroke.

### Search strategy

A search was conducted in PubMed/MEDLINE, Embase, the Cochrane Database of Systematic Reviews, and Web of Science from database inception to 15 January 2026. Search terms combined controlled vocabulary and free-text terms related to MT, AIS, LVO, systematic reviews, and meta-analyses. The PubMed search strategy included combinations of terms related to “mechanical thrombectomy,” “endovascular thrombectomy,” “acute ischemic stroke,” “large vessel occlusion,” “systematic review,” and “meta-analysis.” Equivalent search strategies, adapted to database-specific indexing systems, including EMTREE terminology, were applied in the remaining databases. In addition, the reference lists of included studies and relevant reviews were manually screened to identify additional eligible publications.

### Study selection

**Figure 1.**
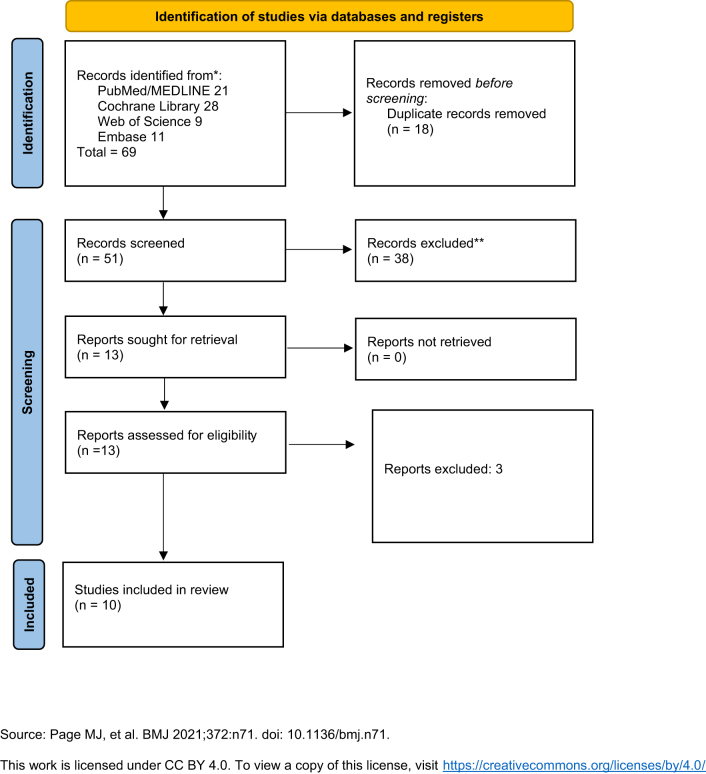
Screening process.

All records were imported into reference management software, and duplicates were removed. Two reviewers independently screened titles and abstracts, followed by a full-text assessment of potentially eligible studies. Disagreements were resolved through discussion and consensus. The study selection process is summarized in Fig. 1.

### Data extraction

Two reviewers independently extracted data using a standardized form. Extracted variables included:

First author and year of publication.

Type of review.

Number and design of included primary studies.

Population characteristics and clinical subgroups.

Interventions and comparators.

Functional, mortality, recanalization, and safety outcomes.

Summary effect estimates, when available.

### Methodological quality assessment

The methodological quality of the included reviews was assessed using AMSTAR-2 (A Measurement Tool to Assess Systematic Reviews). Each review was evaluated across all 16 domains, and overall confidence ratings were reported (Table 1).

**Table 1:** AMSTAR quality assessment.

Study (Authors, Year)	AMSTAR score/quality	Key strengths	Key limitations
Oliveira *et al*, 2022^[^7^]^	9/11	Comprehensive search, meta-analysis of RCTs	Limited to English, heterogeneity in studies
Balami *et al*, 2015^[^8^]^	8/11	RCT focus, pooled ORs	Older devices in some trials
Lambrinos *et al*, 2016^[^9^]^	8/11	Risk of bias assessed	Only 5 RCTs, small sample
Matsumoto *et al*, 2022^[^10^]^	9/11	Network meta-analysis	Moderate heterogeneity in direct vs indirect comparisons
Zheng *et al*, 2023^[^11^]^	10/11	Large number of studies, subgroup analysis	Observational studies included
Toruno *et al*, 2024^[^12^]^	7/11	Focused high-risk population	Small number of studies, heterogeneity
Amoukhteh *et al*, 2023	7/11	IHOS-specific	Limited generalizability, few studies
Liu *et al*, 2022	8/11	Wake-up stroke specific	Small sample sizes
Hou *et al*, 2022^[^13^]^	7/11	Mild stroke subgroup	Observational biases
Adachi *et al*, 2025^[^14^]^	9/11	Anticoagulated population	Only recent studies, heterogeneous anticoagulation regimens

### Data synthesis

Overlap among included reviews was assessed using the Corrected Covered Area (CCA). CCA values were interpreted as slight (<10%), moderate (11–15%), high (16–25%), or very high (>25%). The calculated CCA was 3.4%, indicating slight overlap among reviews. Findings were synthesized narratively and organized according to four domains: functional outcomes, mortality, recanalization success, and safety outcomes, including symptomatic intracranial hemorrhage (sICH). Results were summarized using evidence tables and thematic synthesis to identify areas of consistency, heterogeneity, and evidence gaps across the literature.

## Results

### Study characteristics

Ten systematic reviews and meta-analyses^[^7–16^]^, published between 2015 and 2025, were included in this umbrella review. The reviews synthesized between 5 and 55 primary studies, with total sample sizes ranging from approximately 974 to 20 000 participants. Most reviews evaluated patients with AIS undergoing MT, including clinically important subgroups such as wake-up stroke, in-hospital onset stroke (IHOS), thrombocytopenic patients, and anticoagulated patients. Five reviews included only RCTs, four combined RCTs and observational studies, and one was based predominantly on observational evidence. Detailed study characteristics are presented in Table 2.

**Table 2:** Study characteristics.

Study (Authors, Year)	Type of review	Number of primary studies	Population/Subgroup	Intervention(s)	Comparison	Key outcomes
Oliveira *et al*, 2022^[^7^]^	Systematic review + meta-analysis	15 studies	Acute ischemic stroke (general)	MT + standard care	Standard care only	Functional independence, mortality, recanalization, sICH
Balami *et al*, 2015^[^8^]^	Systematic review + meta-analysis	8 RCTs	AIS with large vessel occlusion	Endovascular thrombectomy (EVT)	Best medical care	mRS 0–2, mortality, sICH
Lambrinos *et al*, 2016^[^9^]^	Systematic review	5 RCTs	AIS with proximal intracranial occlusion	MT ± IVT	IVT alone/best medical therapy	mRS 0–2, mortality, sICH
Matsumoto *et al*, 2022^[^10^]^	Systematic review + network meta-analysis	11 RCTs	Acute stroke	Direct MT, MT + tPA, tPA alone	Comparison among three groups	mRS 0–2, mortality, sICH, successful revascularization
Zheng *et al*, 2023^[^11^]^	Systematic review + meta-analyses	55 studies (9 RCTs + 46 obs)	AIS	MT + IVT	MT alone	FI, excellent outcomes, SR, sICH, aICH, mortality
Toruno *et al*, 2024^[^12^]^	Systematic review + meta-analysis	5 studies	AIS with thrombocytopenia	MT	Non-thrombocytopenic AIS patients	FI, mortality, sICH, recanalization
Amoukhteh *et al*, 2023	Systematic review + meta-analysis	9 studies	In-hospital onset stroke	MT	Community-onset stroke	mRS 0–2, mortality, recanalization, sICH
Liu *et al*, 2022	Systematic review + meta-analysis	12 studies	Wake-up stroke	MT	None/Standard care	mRS 0–2, mortality, sICH, TICI 2b/3
Hou *et al*, 2022^[^13^]^	Systematic review + meta-analysis	11 studies	Mild AIS with LVO	MT	Medical management	Functional prognosis (mRS 0–2)
Adachi *et al*, 2025^[^14^]^	Systematic review + meta-analysis	15 studies	AIS patients on anticoagulants (VKA/DOAC)	MT	Non-anticoagulated patients	FI, mortality, sICH, successful reperfusion
**Study (Authors, Year)**	**Functional Independence (mRS 0–2)**	**Mortality**	**Recanalization/TICI 2b–3**	**Symptomatic ICH**	**Other key findings**
Oliveira *et al*, 2022^[^7^]^	45.6% vs 27.5% (RR 1.65, *P* < 0.01)	16.8% vs 20.1% (RR 0.85, *P* = 0.04)	76.2% vs 33.9% (RR 2.20, *P* < 0.01)	4.78% vs 3.88% (NS)	MT + standard care safe and effective
Balami *et al*, 2015^[^8^]^	OR 1.56, *P* < 0.00001	OR 0.84, *P* = 0.12	N/A	OR 1.03, *P* = 0.88	Newer generation devices increased favorable outcomes
Lambrinos *et al*, 2016^[^9^]^	OR 2.39 (95% CI 1.88–3.04)	NS	NS	NS	MT significantly improves functional independence
Matsumoto *et al*, 2022^[^10^]^	Direct MT ~ MT + tPA (RR 1.02)	RR 1.05, NS	1.60 vs tPA alone	RR 0.83, NS	Direct MT alone acceptable outcomes
Zheng *et al*, 2023^[^11^]^	FI OR 1.27–1.36 (depending on analysis)	Mortality reduced OR 0.65–0.73	SR OR 1.23	sICH OR 1.16–1.24	MT + IVT improved prognosis without increasing HT risk
Toruno *et al*, 2024^[^12^]^	OR 0.83 vs normal (*P* = 0.03)	OR 1.76 (*P* < 0.001)	OR 0.94, NS	OR 1.20, NS	Thrombocytopenic patients had worse FI and mortality
Amoukhteh *et al*, 2023	35.46% vs 40.74%	26.29% vs 18.08%	79.32% vs 81.44%, NS	6.24% vs 6.88%, NS	IHOS worse outcomes than COS, similar recanalization
Liu *et al*, 2022	46.2%	20.4%	83.5%	8.3%	Wake-up stroke MT favorable functional outcomes
Hou *et al*, 2022^[^13^]^	RR 1.01–1.07, NS	N/A	N/A	N/A	MT not significantly associated with better functional outcomes in mild AIS
Adachi *et al*, 2025^[^14^]^	Worse outcomes in anticoagulated (esp VKA)	Higher in VKA (OR 1.61)	Comparable	sICH higher in VKA	DOACs safer than VKA for EVT

DOAC, direct oral anticoagulant; FI, functional independence; SR, successful reperfusion; TICI, thrombolysis in cerebral infarction.

### Quality assessment

Methodological quality, assessed using AMSTAR-2, demonstrated generally robust evidence. Four reviews were rated as high quality, and six as moderate quality. No review was rated as low or critically low quality. Quality assessment findings are summarized in Table 1.

### Functional outcomes

Improved functional independence was the most consistently reported benefit of MT. Oliveira *et al*^[^7^]^ demonstrated significantly higher rates of functional independence with MT compared with standard medical therapy (45.6% vs. 27.5%; risk ratio [RR] 1.65, *P* < 0.01). Similar findings were reported by Balami *et al*^[^8^]^ (odds ratio [OR] 1.56, *P* < 0.00001), Lambrinos *et al*^[^9^]^ (OR 2.39, 95% confidence interval [CI] 1.88–3.04), and Liu *et al*^[^16^]^, who reported favorable outcomes in patients with wake-up stroke.

Evidence comparing treatment strategies was less consistent. Matsumoto *et al*^[^10^]^ found no significant difference in functional independence between direct endovascular thrombectomy (EVT) and EVT plus intravenous thrombolysis (IVT) (RR 1.02, 95% CI 0.88–1.19), whereas Zheng *et al*^[^11^]^ reported superior outcomes with MT plus IVT across RCT-based, observational, and adjusted analyses (ORs 1.27–1.36).

Several subgroup analyses identified populations with poorer outcomes despite MT. Toruno *et al*^[^12^]^ reported reduced functional independence among thrombocytopenic patients (OR 0.83, 95% CI 0.71–0.98), while Amoukhteh *et al*^[^15^]^ found lower rates of functional independence in patients with IHOS compared with community-onset stroke (COS) (35.46% vs. 40.74%, *P* < 0.01). Adachi *et al*^[^14^]^ similarly reported worse outcomes among anticoagulated patients, particularly those receiving vitamin K antagonists (VKA).

The evidence consistently supports MT as an effective intervention for improving functional independence in AIS, although outcomes appear less favorable in specific high-risk subgroups.

### Mortality

Most reviews suggested a mortality benefit associated with MT. Oliveira *et al*^[^7^]^ reported a significant reduction in mortality with MT plus standard medical treatment compared with medical treatment alone (16.8% vs. 20.1%; RR 0.85, *P* = 0.04). Balami *et al*^[^8^]^ also observed a trend toward lower mortality (OR 0.84, *P* = 0.12), while Zheng *et al*^[^11^]^ reported reduced mortality in adjusted analyses of MT plus IVT (OR 0.65, 95% CI 0.49–0.88).

Conversely, mortality remained elevated in certain clinical subgroups. Toruno *et al*^[^12^]^ reported higher mortality among thrombocytopenic patients (OR 1.76, 95% CI 1.26–2.45). Amoukhteh *et al*^[^15^]^ found higher follow-up mortality among patients with IHOS compared with COS (26.29% vs. 18.08%, *P* < 0.01), and Adachi *et al*^[^14^]^ reported increased 90-day mortality among VKA users (OR 1.61).

The evidence suggests that MT is associated with reduced mortality overall, although important disparities remain across vulnerable patient populations.

### Recanalization success

Successful recanalization was consistently improved with MT. Oliveira *et al*^[^7^]^ reported significantly higher revascularization rates following MT compared with standard therapy (76.2% vs. 33.85%; RR 2.20, *P* < 0.01). Similarly, Matsumoto *et al*^[^10^]^ demonstrated superior revascularization with direct EVT compared with thrombolysis alone (RR 1.60, 95% CI 1.33–1.93). Amoukhteh *et al*^[^15^]^ found comparable recanalization rates between IHOS and COS patients undergoing MT (79.32% vs. 81.44%, *P* = 0.11), suggesting that differences in clinical outcomes between these groups may not be explained by technical success alone.

### Safety outcomes

The safety profile of MT was generally favorable, although findings regarding hemorrhagic complications varied across reviews. Oliveira *et al*^[^7^]^ found no significant difference in sICH between MT and comparator groups (RR 1.27, 95% CI 0.88–1.83, *P* = 0.21). Similar findings were reported by Balami *et al*^[^8^]^ and Toruno *et al*^[^12^]^. However, Zheng *et al*^[^11^]^ reported increased risks of both sICH (OR 1.16, 95% CI 1.11–1.21) and asymptomatic intracranial hemorrhage (aICH) (OR 1.24, 95% CI 1.05–1.46) in crude analyses of MT plus IVT. Amoukhteh *et al*^[^15^]^ found no significant difference in sICH between IHOS and COS patients (6.24% vs. 6.88%, *P* = 0.67), whereas Adachi *et al*^[^14^]^ reported significantly higher sICH rates among patients receiving VKA therapy.

Most reviews did not identify a substantial increase in hemorrhagic complications associated with MT. Nevertheless, anticoagulated patients and those receiving combined reperfusion strategies may represent higher-risk populations requiring careful clinical consideration.

### Evidence gaps identified

Despite consistent evidence supporting MT for functional recovery, mortality reduction, and successful recanalization, several knowledge gaps have emerged. Evidence remains limited and largely observational for thrombocytopenic patients, anticoagulated patients, and individuals with IHOS^[^12,14,15^]^. Additionally, conflicting findings persist regarding the comparative effectiveness of direct EVT versus EVT combined with IVT^[^10,11^]^. Safety outcomes, particularly hemorrhagic complications, are inconsistently reported across reviews, highlighting the need for further high-quality studies in clinically complex patient populations.

## Discussion

This umbrella review synthesized evidence from ten systematic reviews and meta-analyses evaluating the efficacy and safety of MT for AIS in the general population and across clinically relevant subgroups. Overall, the included reviews had high- tomoderate-methodological quality according to the AMSTAR-2 assessment. The collective evidence consistently demonstrates that MT is associated with improved functional outcomes, as reflected by higher rates of functional independence (modified Rankin Scale [mRS] score 0–2), compared with standard medical therapy or IVT alone in eligible patients with LVO. In contrast, mortality outcomes were more heterogeneous, with some reviews reporting reduced mortality and others showing no significant difference, particularly when stratified by patient characteristics and clinical context^[^9^]^.

The effectiveness of MT in AIS due to LVO has been firmly established by landmark RCTs, including MR CLEAN, ESCAPE, REVASCAT, SWIFT PRIME, and EXTEND IA, which collectively comprise the HERMES collaboration. These trials demonstrated the superiority of MT over standard medical therapy for anterior circulation LVO^[^17^]^. The findings of the present umbrella review are consistent with this foundational evidence, reinforcing MT as the standard of care for appropriately selected patients with AIS due to LVO.

Safety outcomes across the included reviews suggest that MT is generally safe, with no consistent increase in the risk of sICH compared with medical therapy alone. Nevertheless, hemorrhagic complications, including symptomatic and aICH, post-reperfusion hemorrhage, cerebral edema, and access-site complications, remain clinically relevant concerns. sICH is associated with worse clinical outcomes and may be influenced by factors such as infarct size, elevated serum glucose levels, and device-related vascular injury, including the use of stent retrievers. These complications are thought to arise from mechanical vessel trauma and the subsequent disruption of the blood–brain barrier.

Several reviews explored factors that may modify the effectiveness and safety of MT. Treatment timing remains an important consideration; evidence comparing early (<6 hours) versus extended (>6 hours) symptom-to-puncture windows suggests similar functional outcomes and safety profiles when patients are appropriately selected using advanced imaging techniques. Although differences in sICH and mortality were observed between early and extended treatment windows, these differences did not reach statistical significance after multivariable adjustment^[^18^]^. Age also emerged as an important modifier of outcome, with patients aged 80 years and older demonstrating lower functional independence and higher mortality at 3 months following MT, likely reflecting a higher burden of comorbidities. Notably, advanced age did not significantly affect procedural times or recanalization success rates^[^19^]^.

MT has proven particularly effective in treating large intracranial vessel occlusions, including in patients with lower National Institutes of Health Stroke Scale (NIHSS) scores. In contrast, proximal occlusions remain associated with a poorer prognosis^[^20^]^. Evidence further suggests that MT is most effective when combined with standard medical treatment, including IVT in eligible patients, and when thrombus characteristics are favorable, such as shorter clot length^[^20^]^. Variability in patient characteristics, occlusion location, stroke severity, treatment timing, and adherence to guideline-based care contributes to heterogeneity in outcomes across studies.

Advances in neuroimaging have expanded the therapeutic window for MT to as long as 24 hours in carefully selected patients with salvageable brain tissue identified by computed tomography and magnetic resonance imaging. In parallel, emerging biomarkers, such as glial fibrillary acidic protein (GFAP), have shown potential utility for diagnosing and prognosticating AIS. GFAP, an intermediate filament protein expressed in astrocytes, is typically absent from the circulation but becomes elevated following ischemic brain injury, with serum levels correlating with stroke severity and NIHSS scores during follow-up^[^21^]^. While promising, the clinical integration of such biomarkers requires further validation.

Early recognition of stroke symptoms and rapid referral to specialized stroke centers remain critical to maximizing the benefits of MT. Clinical decision-making should incorporate patient age, stroke severity, comorbidities, occlusion characteristics, and treatment timing, particularly in borderline or high-risk subgroups. Further high-quality research is needed to better define the efficacy and safety of MT in these populations and to refine patient selection strategies that optimize outcomes while minimizing complications.

The evidence synthesized in this umbrella review demonstrates several important strengths. MT consistently improves functional independence and recanalization rates across multiple systematic reviews and meta-analyses. These findings are supported by high-quality RCTs and reinforced by real-world observational studies. Additionally, the overall safety profile of MT is favorable, with most reviews reporting no significant increase in sICH compared with standard medical therapy. The benefits of MT have been demonstrated across a variety of clinical settings, supporting its role as a cornerstone treatment for AIS due to LVO. Despite these strengths, important challenges remain. Evidence for several clinically relevant populations, including thrombocytopenic patients, anticoagulated patients, and individuals with IHOS, is limited and relies largely on observational data. Furthermore, conflicting findings regarding direct MT versus bridging therapy indicate that the optimal reperfusion strategy remains uncertain. Variability in patient selection criteria, treatment timing, stroke severity, and outcome reporting also contributes to heterogeneity across studies, making direct comparisons difficult. These gaps highlight the need for further high-quality research to refine patient selection and optimize treatment strategies.

This umbrella review provides clinicians with a consolidated summary of the highest available evidence regarding the efficacy and safety of MT. The findings support the continued use of MT in appropriately selected patients, while emphasizing the importance of individualized decision-making in high-risk populations. For researchers, this review identifies key evidence gaps and research priorities, particularly in understudied patient subgroups and unresolved treatment strategies.

## Limitations

Several limitations of this umbrella review should be acknowledged. As with all umbrella reviews, conclusions are constrained by the quality and scope of the underlying systematic reviews and meta-analyses rather than the primary studies directly. The CCA analysis revealed slight overlap (3.4%) among included reviews, indicating that some primary studies contributed to multiple meta-analyses; while minimal, this may introduce a degree of double-counting in the narrative synthesis. Additionally, the included reviews employed heterogeneous effect measures (ORs and RRs) and varied definitions for key outcomes, such as functional independence and recanalization success, limiting direct cross-review comparability.

## Conclusion

This umbrella review synthesizes evidence from high- to moderate-quality systematic reviews and meta-analyses evaluating MT for AIS. Overall, the findings support the efficacy of MT in improving functional independence and achieving higher rates of successful recanalization compared with standard medical therapy or IVT alone in eligible patients with LVO. Although mortality and safety outcomes were more heterogeneous across reviews, MT was generally associated with a favorable benefit-risk profile and was not consistently linked to an increased risk of sICH. This review highlights important areas of uncertainty, particularly in thrombocytopenic patients, anticoagulated patients, and individuals with IHOS, as well as ongoing questions regarding optimal reperfusion strategies. These findings emphasize the need for individualized clinical decision-making that incorporates patient characteristics, stroke severity, treatment timing, and imaging findings. For clinicians, this umbrella review provides a concise synthesis of the highest available level of evidence to support treatment decisions in AIS. For researchers, it identifies key evidence gaps and priorities for future investigation, including understudied patient populations and the optimization of treatment pathways.

## Data Availability

Data sharing is not applicable to this article, as no datasets were generated or analyzed during the current study.
